# Sequential CAR T cell and targeted alpha immunotherapy in disseminated multiple myeloma

**DOI:** 10.1007/s00262-023-03461-z

**Published:** 2023-05-20

**Authors:** Dennis Awuah, Megan Minnix, Enrico Caserta, Theophilus Tandoh, Vikram Adhikarla, Erasmus Poku, Russell Rockne, Flavia Pichiorri, John E. Shively, Xiuli Wang

**Affiliations:** 1 Department of Hematology, City of Hope Medical Center, Beckman Research Institute, Duarte, CA 91010 USA; 2 Department of Immunology and Theranostics, Beckman Research Institute, City of Hope, Duarte, CA 91010 USA; 3 Department of Hematologic Malignancies Research Institute, City of Hope Medical Center, Duarte, CA 91010 USA; 4 Division of Mathematical Oncology and Computational Systems Biology, Beckman Research Institute, City of Hope, Duarte, CA 91010 USA; 5 City of Hope Medical Center, Duarte, CA 91010 USA

**Keywords:** Multiple myeloma, CAR T therapy, Targeted alpha therapy

## Abstract

Multiple myeloma (MM) is still an incurable disorder despite improved antibody and cellular therapies against different MM antigens. Single targeted antigens have so far been ineffective against MM with most patients relapsing after initial response. Hence, sequential immunotherapies directed at different targets are expected to perform better than monotherapy alone. Here, we optimized and established in preclinical studies the therapeutic rationale of using targeted alpha therapy (TAT) directed against CD38 antigen (^225^Ac-DOTA-daratumumab) with CAR T cell therapy directed at CS1 antigen in a systemic MM model. The sequential therapies compared CAR T therapy followed by TAT to TAT followed by CAR T therapy. CAR T cell monotherapy increased median survival from 49 days (d) in untreated controls to 71d with a modest improvement to 89d for 3.7 kBq of TAT given 14d later. When CAR T was followed by 7.4 kBq of TAT 29d later, sequential therapy increased median survival from 47d in untreated controls to 106d, compared to 68d for CAR T monotherapy. When CAR T therapy was followed by *untargeted* alpha immunotherapy using 7.4 kBq of ^225^Ac-DOTA-trastuzumab (anti-HER2) antibody 29d later, there was only a slight improvement in response over CAR T monotherapy demonstrating the role of tumor targeting. TAT (7.4 kBq) followed by CAR T therapy was also effective when CAR T therapy was delayed for 21d vs 14d or 28d post TAT, highlighting the importance of timing sequential therapies. Sequential targeted therapies using CS1 CAR T or ^225^Ac-DOTA-CD38 TAT in either order shows promise over monotherapies alone.

**Supplementary Information:**

The online version contains supplementary material available at 10.1007/s00262-023-03461-z.

## Introduction

Multiple myeloma (MM), a blood cancer of plasma cells with an incidence of over 30,000 cases and 12,000 deaths per year [1], has been treated with targeted immunotherapies to CS1 [2], BCMA [3], and CD38 antigens [4], but each has met with eventual recurrence. In terms of optimal target selection, CS1 (CD319 or SLAMF7) is highly expressed on > 95% MM cells, and more commonly expressed on MM than BCMA [5], irrespective of genetic abnormalities and disease stage [6]. Therefore, CS1 chimeric antigen receptor (CAR) T cell therapy can be an effective strategy, especially for those MM cases that are BCMA negative. Moreover, CS1 CAR T cells in our studies have demonstrated efficient cytolytic function and potent anti-MM activity in vivo [7], whereas other studies have indicated that CS1 CAR T cells eliminate MM cells by targeting both proximal and distal domains of CS1 [8–10]. Therefore, combining CS1 CAR T cells with other established forms of therapy may boost efficacy against MM tumors and achieve durable remission.

Since recurrences are usually associated with down-regulation of tumor antigen after targeted therapies [11] or incomplete elimination of residual tumor cells, selection of a target antigen that is not down-regulated (such as CS1 and CD38) is an attractive strategy. For example, anti-CD38 Daratumumab (Dara) immunotherapy eventually becomes ineffective due to a lack of CD38 targeted killing, but continued expression of antigen is observed on the MM cells [12], suggesting that arming anti-CD38 antibodies with cytotoxic agents such as radionuclides would enable further treatment. Recently, we [13] and others [14, 15], have shown that targeted alpha therapy (TAT) using ^225^Ac labeled anti-CD38 antibodies (^225^Ac-CD38 TAT) can be an effective and minimally toxic therapeutic strategy to eradicate MM. Although *α*- particles have a limited range in tissue of about 40–100 microns, their high linear energy transfer is more efficient at tumor therapy than longer range *β*-emitters such as ^177^Lu [13]. Since only 30% of patients who progress on Dara + immunomodulatory drug (IMID) regimens (for treatment of relapsed/refractory MM) experience long-term clinical benefits (> 12 months) from CAR T therapies, there is an urgent medical need to develop novel therapeutic interventions to improve the long-term efficacy of MM therapies.

Several active clinical trials are underway to explore the combination of external beam radiation therapy with immunotherapy for improved survival and toxicity control [16, 17]. In this study, we investigate a novel combination approach involving Dara-based radiation therapy and CS1 CAR T cell therapy targeting different MM antigens through unique mechanisms in animal models. Since antigen heterogeneity and antigen down-regulation occur in most malignancies, a strong rationale supports the exploration of sequential therapies that target different antigens on the same tumor. Here, we tested sequential therapies with CS1 CAR T cell therapy or ^225^Ac-CD38 TAT given first or second in an MM model of systemic disease. The rationale is supported by (a) CAR T cell therapies in MM are approved for the treatment of multi-relapsing patients of which the majority have progressed after anti-CD38 based therapies; (b) CD38 remains targetable on the surface of cancer cells in multi-relapsing MM patients [12] and continued clinical targeting of CD38 can further increase the therapeutic options and survival of Dara treated patients. While we test few regimens of these therapies, it is not possible to test every combination. Thus, mathematical modeling [18] is an excellent tool that allows us to test different therapeutic combinations in silico. It informs us about the optimal therapeutic combinations as well as the expected therapeutic response and allows prediction of optimal time intervals between sequential therapies, especially in cases where one of the treatments, namely TAT, may interfere with the other, namely CAR T therapy. In addition, mathematical modeling offers a way to tailor a therapeutic regimen to individual patients based on their tumor growth characteristics. Here we sequentially add ^225^Ac-CD38 TAT to CS1 CAR T therapy at two different intervals to test the hypothesis that two immunotherapies directed at different antigens with different modes of killing would be more effective than that with either monotherapy. Optimized sequential therapy may eliminate residual tumor cells without utilizing toxic high doses of each agent, with the goal of reducing disease relapse. We also compare the sequential administration of CS1 CAR T cell treatment after ^225^Ac-CD38 TAT at three different intervals to demonstrate the critical effect of timing the CAR T therapy for optimal results.

## Materials and methods

### Ethics declaration

All animal studies were performed in accordance with IACUC protocols approved by the City of Hope Institutional Animal Care and Use Committee, and in accordance with the NIH Office of Laboratory Animal Welfare guidelines.

### Antibodies, reagents, and cell lines

Daratumumab (Dara), anti-CD38 antibody, was obtained from Janssen Biotech Inc. (Titusville, NJ). Anti-human EGFR antibody was obtained from Biolegend and human T cell expander CD3/CD28 dynabeads were from Thermofisher Scientific. MM.1S cells were purchased from ATCC and cultured in RPMI medium supplemented with 2 mM L-glutamine and 10% heat-inactivated FBS. In order to generate firefly luciferase (ffluc) green fluorescent protein (GFP +) cell lines, MM.1S cells were transduced with lentiviral vector encoding eGFP-ffluc and GFP positive cells were sorted by FACS to obtain > 98% purity. Aliquots of passaged cells were frozen in cyrostor CS10 (Biolife Solutions) and stored in liquid nitrogen. 1,4,7,10-Tetraazacyclododecane-1,4,7,10-tetraacetic acid mono-N-hydroxysuccinimide ester (DOTA-NHS-ester) was purchased from Macrocyclics, Inc., Plano, TX. ^225^Ac was obtained from the Department of Energy, Oakridge National Laboratory, Oakridge, TN.

### Radiolabeling

Dara or control trastuzumab (Tras) antibodies were reacted with a 30 molar excess of the chelator DOTA-NHS ester as previously described (6). DOTA conjugation was confirmed by Q-TOF mass spectrometry (Agilent Technology 6510 Q-TOF LC/MS) as follows: 6 µg of antibody was reduced with 1 µL of 0.2 M Tris(2-carboxyethyl)phosphine (TCEP) for 2 h at 37 °C and then analyzed on an HPLC protein Agilent chip (Agilent Technologies, Santa Clara, CA). DOTA-conjugated antibody (50 µg) was incubated with ^225^Ac at a labeling ratio of 1.85 MBq/µg for 45 min at 43 °C and chased with 1 mM DTPA. Radiolabeling efficiencies determined by instant thin layer chromatography were between 89 and 100% for all reactions.

### Generation of CS1-specific CAR T cells

Clinical grade CS1 lentiviral vector was constructed consisting of a CS1-specific scFv linked to an intracellular 4-1BB co-stimulatory and CD3ζcytoplasmic domain by a modified IgG4 hinge region, (i.e., deleted CH2 region for enhanced persistence). A truncated human EGFR (huEGFRt) was used as a transduction marker and was separated from the codon optimized CS1:4-1BB: z sequence by a T2A ribosomal skip sequence. Leukapheresis products from healthy human donors were obtained and PBMCs separated by density gradient centrifugation using Ficoll (Amersham Biosciences). Subsequently, T naïve/memory (Tn/mem) cells were isolated by CD62L + microbeads from the resulting negative fraction, following depletion of CD14 + and CD25 + cells (AutoMACS, Miltenyi Biotech). Following selection, Tn/mem cells were activated with CD3/CD28 microbeads, transduced with CS1 lentivirus and expanded as previously described (6). CAR T cells were characterized for CAR percentage based on EGFR expression and banked in liquid nitrogen for animal experiments. All healthy donor samples were obtained under approved COH IRB protocols (IRB 09025).

### Animal studies

All animal studies were performed in NOD.Cg-Prkdcscid Il2rgtm1Wjl/SzJ mice (NSG; 6–10 weeks old; Jackson Laboratory) (IACUC 21034). Animals were housed in pie cages, in a specific pathogen free (SPF) room, with a maximum of 5 mice per cage. Mice were engrafted with 5 × 10^6^ MM.1S eGFP-ffluc lines intravenously (I.V) and randomized into groups 6 days post tumor injection, based on bioluminescence imaging (BLI). On day 7 post tumor engraftment, mice were treated with 1 × 10^6^ CS1 CAR T cells (based on CAR expression) as well as matched number of mock T cells. Untreated groups received PBS. Prior to start of TAT, mice were given IVIg by i.p. injection for 2 h and subsequently treated with saline (untreated group), 3.7 or 7.4 kBq of untargeted ^225^Ac-DOTA-Tras, 3.7 or 7.4 kBq of targeted ^225^Ac-DOTA-Dara either 21- or 36-days post MM1-S injection. All TAT doses were made up to 30 μg antibody, for a total volume of 200 µL and given intravenously. Tumor distribution and growth was followed by serial whole-body imaging on the Lago X (Spectral Instruments Imaging, Tucson, AZ). Before in vivo imaging, animals were anesthetized with 4% isoflurane and injected intraperitoneally with 200 µL D-luciferin (15 mg/ml) in sterile PBS. All BLI data are depicted in radiance units (photons/s/cm2/sr) measured over the whole body as the region of interest. Mice were grouped so that the average initial BLI was similar across all groups. Whole-body toxicity was measured by monitoring weight loss over time of therapy, with weight loss > 20% considered an experimental endpoint. Paralysis of the mouse hind legs, a common symptom of the MM tumor models, was used as an alternative endpoint.

### Statistical analysis

Two-way ANOVA was used to analyze the tumor growth curves, using Prism 9.4.0 (GraphPad Software). The log-rank Mantel-Cox test was used to analyze the survival curves. Each treated group was compared back to the saline control group, unless otherwise stated. Differences were considered significant if *P* ≤ 0.05.

## Results

### Preliminary sequential CAR T cell therapy followed by targeted alpha therapy 14 days later

**Fig. 1:**
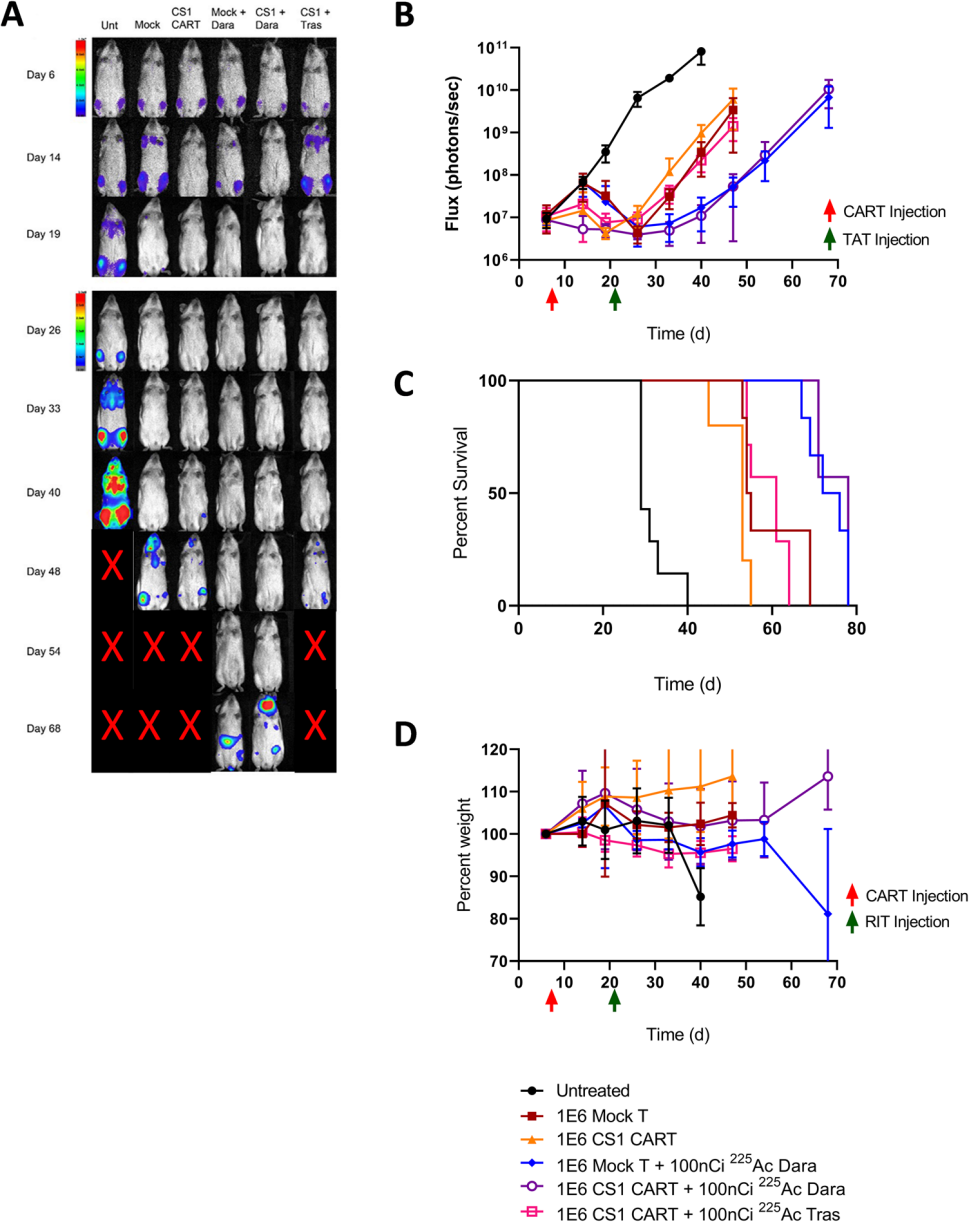
Efficacy of sequential therapy with donor 1 CS1 CAR T and 3.7 kBq TAT 14 days later for treatment of disseminated MM. **A** Representative BLI images for each group, color bar indicating intensity. **B** MM burden as quantified using BLI images, in radiance. (Mock, *P* = 0.011; CS1 CAR T, *P* = 0.012; Mock + Dara, *P* = 0.011; CS1 CAR T + Dara, *P* = 0.011; CS1 CAR T + Tras TAT, *P* = 0.011) **C** Kaplan–Meier survival plot (Mock, *P* = 0.0004;CS1 CAR T, *P* = 0.0011; Mock + Dara, *P* = 0.0004; CS1 CAR T + Dara, *P* = 0.0001; CS1 CAR T + Tras TAT, *P* = 0.0001) **D** Whole-body toxicity as measured by weight (Mock, ns; CS1 CAR T, *P* = 0.0009; Mock + Dara, ns; CS1 + Dara, *P* = 0.042; CS1 CAR T + Tras TAT, ns). *n* = 7 for all groups but Mock /Mock + Dara (*n* = 6) and CS1 CAR T (*n* = 5). Red and green colored arrows represent time of administration of CS1 CAR T and TAT therapy respectively, Time (d) indicates days post tumor engraftment

Sequential CS1 CAR T treatment followed by ^225^Ac-DOTA-CD38 targeted alpha therapy (TAT) was tested in a disseminated MM mouse model by inoculating MM.1S cells intravenously. Since radiation from ^225^Ac-DOTA-CD38 TAT accumulated in the tumor could indirectly affect the anti-MM activity of CS1 CAR T cell viability infiltrated in the tumor site, a preliminary TAT treatment schedule of 14 days post CAR T cell therapy was investigated. CS1 CAR T cell therapy was performed 7 days after tumor engraftment, followed by TAT 14 days later, a time at which tumor regrowth was predicted by CAR T cell monotherapy [7]. An initial activity of 3.7 kBq of ^225^Ac-DOTA-CD38 TAT was chosen based on our previous study in which 3.7 kBq had a therapeutic effect with low off-target toxicity [13]. Mice treated with CS1 CAR T cell monotherapy had a delay in tumor growth of 10 days compared to untreated controls (Fig. 1A–B, Fig. S1) and a median survival of 49 days compared to 40 days for untreated controls (Fig. 1C and Table S1). However, mock therapy with activated T cells had a similar delay in tumor growth and median survival (Fig. 1A–C and Table S1), suggesting that activated T cells from this donor were equipotent to CAR T cells, a phenomenon occasionally observed with some donors [19]. Nonetheless, the addition of ^225^Ac CD38-TAT 14 days later increased the delay in tumor growth to 20 days compared to the untreated controls, or 10 days for CS1 CAR T monotherapy. Sequential CAR T plus TAT increased median survival to 71 vs 49 days for CAR T cell monotherapy with similar results for sequential mock therapy plus TAT. As a further control, untargeted *α*-immunotherapy with ^225^Ac-DOTA-Trastuzumab at 14 days post CS1 CAR T therapy had a negative impact on the CAR T cell therapy, decreasing median survival from 71 to 49 days. Whole-body toxicity was monitored by whole-body weight loss (Fig. 1D). As expected, untreated animals had major weight loss at 30–40 days just prior to succumbing to systemic disease, otherwise the various treated groups had minimal weight loss until they were euthanized based on an upper limit of bioluminescent readings.

### Sequential CAR T therapy followed by TAT 29 days later

**Fig. 2:**
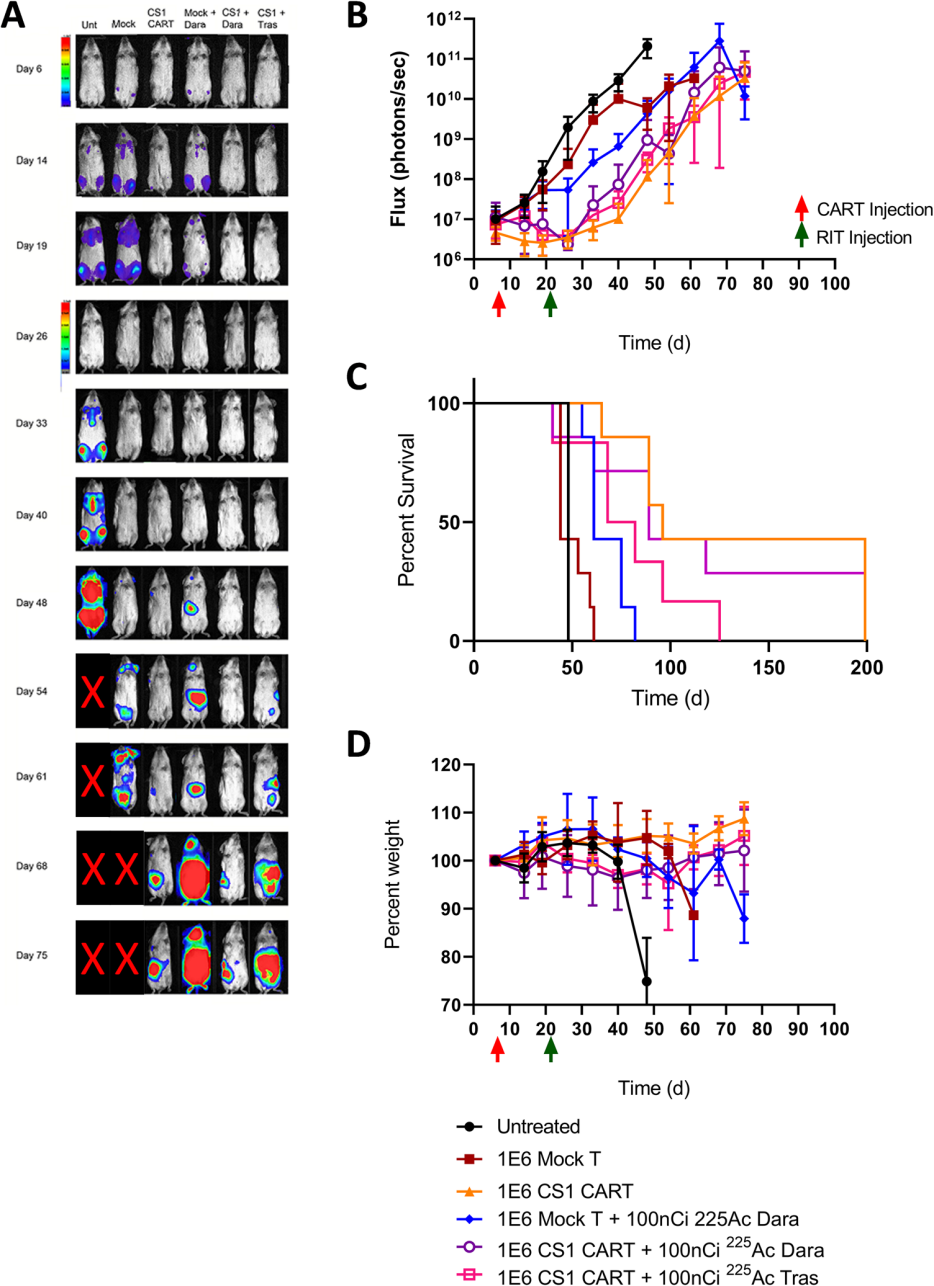
Sequential therapy of disseminated MM by CS1 CAR T from donor 2 plus 3.7 kBq *α*-therapy 14 days later for treatment of disseminated MM. **A** Representative BLI images for each group, color bar indicating intensity (note scale change on day 28). **B** MM burden as quantified using BLI images. **C** Kaplan–Meier survival plot (*P* values vs untreated: Mock, *P* = 0.8076; CS1 CAR T, *P* = 0.0016; Mock + Dara targeted *α*-therapy, *P* = 0.0016; CS1 CAR T + Dara targeted *α*-therapy, *P* = 0.0292; CS1 CAR T + Tras untargeted *α*-therapy, *P* = 0.0629). **D** Whole-body toxicity as measured by percent body weight (P values vs untreated: Mock, ns; CS1 CAR T, *P* = 0.027; Mock + Dara targeted *α*-therapy, *P* = 0.016; CS1 CAR T + Dara targeted *α*-therapy, ns; CS1 CAR T + Tras untargeted *α-*therapy, ns). *N* = 7 for all groups except untreated (*n* = 4) and CS1 CAR T + Tras untargeted *α*-therapy (n = 6). Red and green colored arrows represent time of administration of CAR T and TAT therapy respectively, Time (d) indicates days post tumor engraftment

Based on the results of the preliminary study above, we made two modifications to the study: (a) a second donor for the CS1 CAR T therapy was tested in which mock therapy with activated T cells was minimal, and (b) TAT was delayed from 14 to 29 days post CAR T cell therapy. Twenty-nine days post CS1 CAR T cell therapy was chosen to further lower the risk of radiotoxicity of circulating ^225^Ac-DOTA-CD38 antibodies to persisting CAR T cells. As expected with this donor, tumor growth delay and survival with mock therapy with activated T cells was similar to the untreated controls (Fig. 2A–C, Fig. S2, Table S1). CS1 CAR T cell monotherapy led to a delay of tumor growth of 28 days compared to untreated controls and an increase in median survival of 96 days compared to 48 days for untreated controls. There was a small effect of mock therapy plus TAT on median survival (61 days) that was much less than CS1 CAR T cell therapy plus TAT (89 days). Interestingly, untargeted alpha therapy with ^225^Ac-DOTA-Trastuzumab had a slight effect on tumor growth inhibition compared to targeted ^225^Ac-DOTA-CD38, but a lower median survival (75 days) compared to CS1 CAR T plus TAT (89 days). In terms of whole-body toxicity, none of the treatments resulted in significant weight loss (Fig. 2D).

**Fig. 3:**
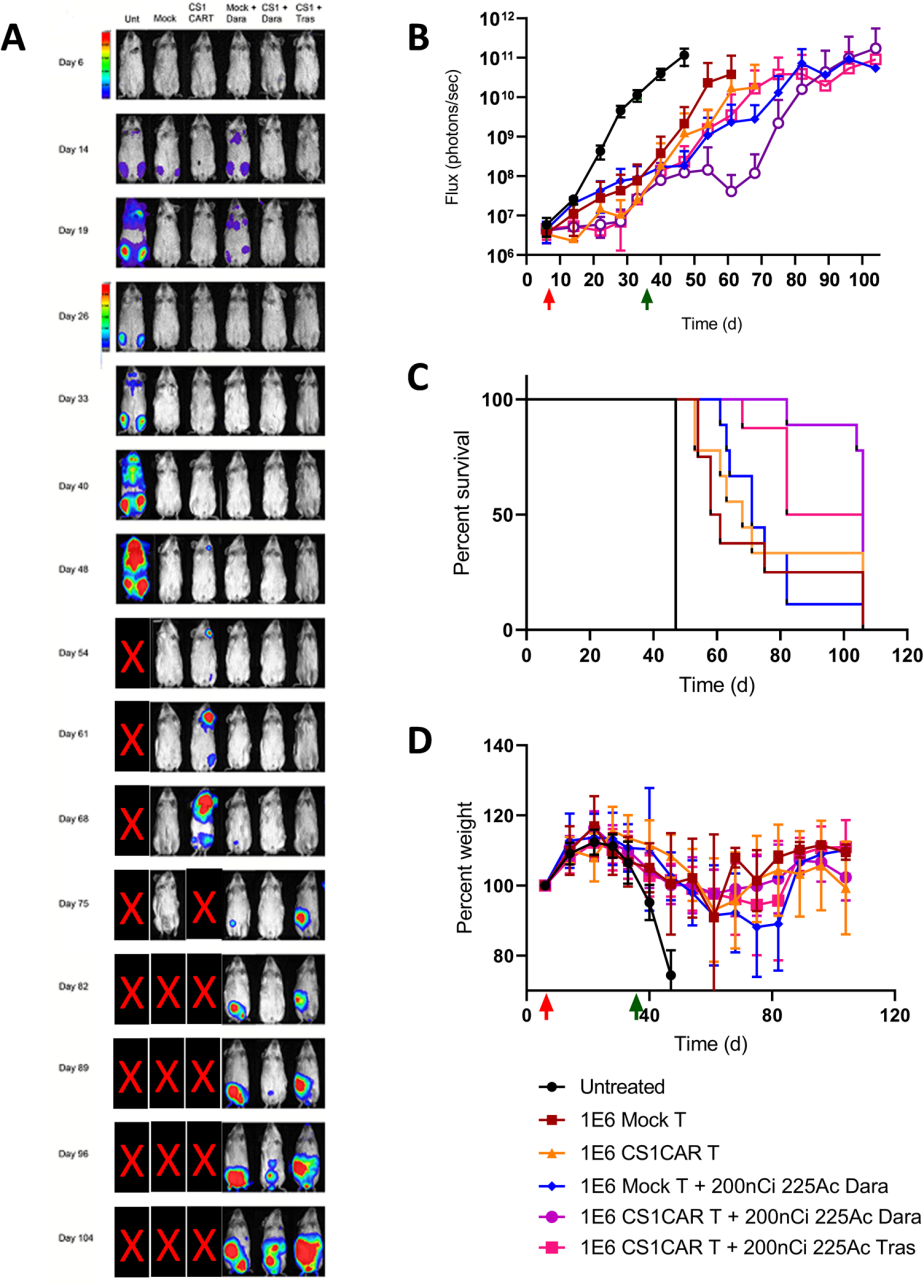
Sequential therapy of disseminated MM by CS1 CAR T from donor 2 plus 7.4 kBq *α-*therapy 29 days later for treatment of disseminated MM. **A** Representative BLI images for each group, color bar indicating intensity (note scale change at day 28). **B** MM burden as quantified using BLI images, in radiance. (P values vs untreated: Mock, *P* = 0.0009; CS1 CAR T, *P* = 0.0008; Mock + Dara targeted *α*-therapy, *P* = 0.0007; CS1 CAR T + Dara targeted *α*-therapy, *P* = 0.0007; CS1 CAR T + Tras untargeted *α*-therapy, *P* = 0.0007) **C** Kaplan–Meier survival plot. (*P* values vs untreated: Mock, *P* = 0.0001; CS1 CAR T, *P* < 0.0001; Mock + Dara targeted *α*-therapy, P < 0.0001; CS1 CAR T + Dara targeted *α*-therapy, *P* < 0.0001; CS1 CAR T + Tras untargeted *α*-therapy, *P* = 0.0001) (**D**) Whole-body toxicity as measured by percent body weight (P values vs untreated: Mock, ns; CS1 CAR T, *P* = 0.0004; Mock + Dara targeted *α*-therapy, *P* = 0.0026; CS1 CAR T + Dara targeted *α-*therapy, *P* = 0.010; CS1 CAR T + Tras untargeted *α*-therapy, ns). Untreated, CS1 CAR T + untargeted *α*-therapy Tras (N = 8); Mock, CS1 CAR T (N = 6); Mock + targeted *α*-therapy Dara, CS1 CAR T + targeted *α*-therapy Dara (N = 9). Red and green colored arrows represent time of administration of CAR T and TAT therapy respectively, Time (d) indicates days post tumor engraftment

The surprising efficacy of untargeted vs targeted alpha therapy was further explored by increasing the dose of alpha therapy from 3.7 to 7.4 kBq to test the effect of a higher tumor dose of TAT vs the deleterious effect of circulating radiolabeled antibody on toxicity. Importantly, we had previously shown that a dose of 7.4 kBq ^225^Ac-DOTA-CD38 monotherapy had low whole-body toxicity [13]. In the 7.4 kBq repeat study, the tumor growth delay of CS1 CAR T cell monotherapy was similar (28 days) to the previous study, but sequential CAR T therapy plus TAT had an increase in median survival of 106 days compared to 94 days for CAR T plus untargeted alpha therapy (Fig. 3A–C, Fig. S3, Table S1). Thus, there was a major improvement in sequential CAR T therapy followed by an increased dose of TAT with a lesser effect of untargeted alpha therapy at the same dose. Whole-body toxicity as measured by weight loss was minimal for the single or combined therapy groups (Fig. 3D).

### Sequential ^225^Ac-DOTA-CD38-TAT followed by CAR T cell treatment

**Fig. 4:**
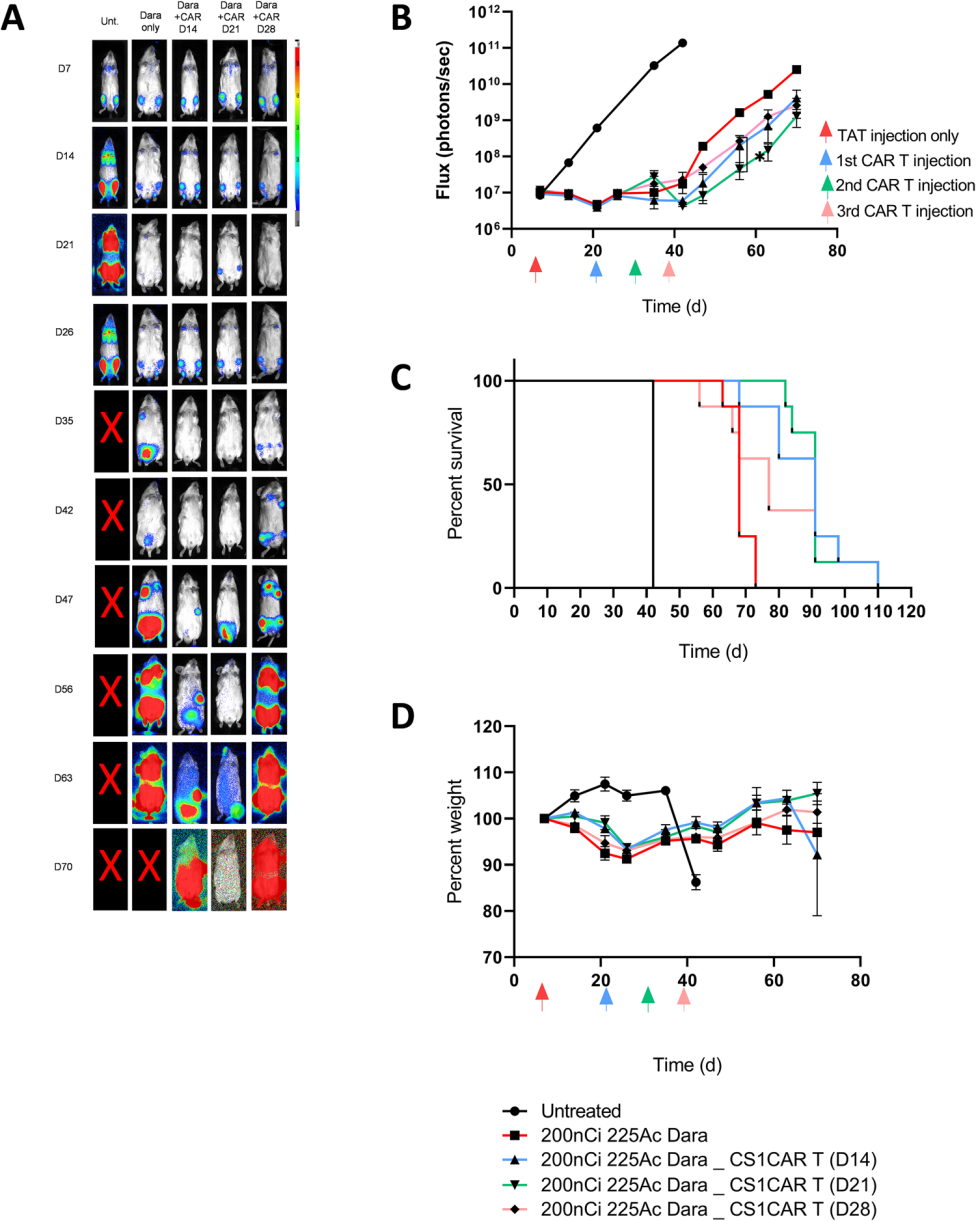
Sequential therapy of disseminated MM by 7.4 kBg of ^225^Ac-CD38 TAT and CAR T cell administered at different time points. **A** Representative BLI images for each group, color bar indicating intensity. **B** MM burden as quantified using BLI images, in radiance. (P values for day 21 vs day 28 CS1 CAR T cells on week 8: *P* = 0.0169. *P* values vs untreated: Dara targeted α therapy only, P < 0.0001, Dara targeted α therapy + CS1 CAR T (D21), P < 0.0001, Dara targeted α therapy + CS1 CAR T (D28), *P* < 0.0001, Dara targeted α therapy + CS1 CAR T (D35), *P* < 0.0001 **C** Kaplan–Meier survival plot. (*P* values vs untreated: Dara targeted α therapy only, *P* = 0.0001, Dara targeted α therapy + CS1 CAR T (D 14), *P* = 0.0001, Dara targeted α therapy + CS1 CAR T (D 21), *P* = 0.0001, Dara targeted α therapy + CS1 CAR T (D 28), *P* = 0.0001. **D** Whole-body toxicity as measured by percent body weight (*N* = 8). Colored arrows represent time of administration of TAT or CAR T therapy at indicated days, Time (d) indicates days post tumor engraftment

In the above studies, we explored CAR T cell therapy followed by TAT. To determine whether sequential treatment of ^225^Ac-DOTA-CD38 TAT first followed by CS1 CAR T therapy would yield equivalent or better tumor growth inhibition and/or median survival compared to CS1 CAR T before TAT, we treated mice with disseminated MM with 7.4 kBg of ^225^Ac-DOTA-CD38 TAT followed by CS1 CAR T cell treatments at 14, 21 and 28 days post TAT. The different intervals were chosen to determine if the residual radioactivity of radiolabeled antibody would have a deleterious effect on the subsequently administered CAR T cells. The timing of a second treatment was predicted to be crucial by mathematical modeling [18] in which no improvement in progression-free survival would be achieved once tumor regrowth reached a critical stage regardless of the order of the two therapies. As shown in Fig. 4, tumor growth delay was best for mice receiving CS1 CAR T cells 21 days post ^225^Ac-CD38 TAT (28 days post tumor engraftment) with an overall survival of 91 days compared to 42 days for untreated controls (Fig. 4A–C, Fig. S4, Table S1). Timing of the CS1 CAR T cell therapy at 28 days likely occurred too late for the CAR T therapy to control increased tumor burden (median survival of 77 days). Whole-body toxicity as measured by weight loss (Fig. 4D) was transient and recovered post therapy.

## Discussion

The high toxicity of treatment regimens that limit the maximum dose is a major reason for occurrence of residual tumor as causes of MM relapse. Sequential immunotherapies with CS1 CAR T cells and ^225^Ac CD38-TAT were chosen to increase the potency to toxicity ratio and to achieve a more durable remission by targeting two highly expressed MM antigens with different cytotoxic mechanisms. We found that the timing of either therapy, CS1 CAR T followed by ^225^Ac-DOTA-CD38-TAT or TAT followed by CAR T therapy, was critical to delay the effects of circulating ^225^Ac radiolabeled antibody on CAR T therapy. In the first case, CS1 CAR T followed by ^225^Ac-DOTA-CD38-TAT performed better when TAT was delayed for 29 vs 14 days and the dose of TAT was increased from 3.7 kBq to 7.4 kBq. In the second case, delay of CS1 CAR T therapy for 21 days post TAT performed better than a delay of 14 or 28 days. Non-targeting TAT (^225^Ac-DOTA-trastuzumab) served as an additional control to ensure specificity of sequential therapies, but was excluded in later studies (i.e., TAT followed by CAR T) based on data from preceding experiments (Figs. 1, 2, 3). Other approaches to improve CAR T therapy include sequential treatment with CAR T plus checkpoint inhibitors such as anti-PD-1 or anti-PD-L1 [20, 21]. For example, in the treatment of a diffuse B-cell lymphoma that progressed on CD19 CAR T therapy, subsequent treatment with anti-PD-L1 therapy led to regression of multiple lesions [20]. A safety study of neuroblastoma with CAR T plus anti-PD-1 therapy [21] suggested that this approach may be a safe option for combination immunotherapies of this kind. In addition, combination therapy by cell intrinsic approaches has been described [22]. In this approach the CAR T cells are engineered to secrete scFv antibodies to checkpoint inhibitors. For example, CD19 CAR T cells engineered to secrete a scFv anti-PD-1 antibody led to higher survival of mice engrafted with CD19 positive ID8 tumors that expressed PD-1 on their CAR T cells [22]. In comparison, the main advantage of our approach was that each therapy (whether CAR T or targeted α-immunotherapy) was tumor targeted and to distinct tumor antigens.

In vivo persistence of CAR T cells has a major effect on timing of a second therapy. Thus, optimal timing of ^225^Ac CD38-TAT was at the point of tumor regrowth when CS1 CAR T cell therapy was no longer effective. Since activated T cells, including CS1 CAR T cells, express high levels of CD38 (Fig. S5), ^225^Ac CD38-TAT treatment given at an early time point may eliminate persisting CS1 CAR T cells. When tumor burden was used to parameterize CAR T cell therapy, we found that tumor proliferation rate was vital to determining CAR T and TRT administration [18]. Thus, the window for the administration of ^225^Ac CD38-TAT can be mathematically modeled to achieve maximum effects of the sequential therapy [18]. So far, the window for timing sequential immunotherapies has not been thoroughly explored.

The rationale in targeting two different antigens in MM by sequential targeted therapies was demonstrated, including showing that the order of sequential therapies was similarly effective, as long as the timing between the two modalities was optimal. We believe that mathematical modeling studies will help in developing better therapy regimens and reveal the mechanisms behind them [18]. In our mathematical modeling work earlier, we have incorporated the deleterious effect of TAT on CAR T cells using a radiosensitivity parameter. Thus, simultaneous presence of both TAT and CAR T cells has a negative impact on the therapeutic efficacy due to CAR T cells being unable to show full potency. On the other hand, an increased tumor burden also adversely impacts TAT or CAR T cell response due to increased tumor-to-therapeutic ratio. Thus, the parameterization of the mathematical model based on the experimental data is an important step for development of an optimal therapeutic regimen when combining two therapies. In fact, this proof-of-concept study could be generalizable for sequential CAR T therapy and TAT against MM with the many targeting antigens and therapies that are now available. A critical issue, however, to consider in combination therapies involving CAR T cell and radiation therapies is toxicity. Toxicity in CAR T cell therapy is typically shown in the form of a cytokine release syndrome that limits the dose of the therapeutic agent administered. Our data provide a strong rationale for better therapy without increasing the dosage of CAR T cells and radiation, but meanwhile demonstrates that a careful exploration in issues of dosing and timing is likely required in clinical settings.

## Conclusion

Sequential therapies with CAR T plus TAT directed and different targets on the same tumor in either order are similarly effective as long as the interval between the two therapies is optimized to tumor regrowth. Although untargeted alpha therapy has some beneficial effect on systemic disease, there was a substantial increased efficacy for targeted alpha therapy. In terms of whole-body toxicity, the limited tissue penetration of alpha particles is an advantage, at least in systemic therapy. Thus, these results underscore the importance of sequential targeted therapies with different targeting mechanisms, emphasizing how TAT may play a special role.

## Supplementary Information

Below is the link to the electronic supplementary material.

Supplementary file1 (DOCX 6470 kb)

## Data Availability

The data generated in this study are available in the article and the supplementary data files.
