# Supplementary material for: Sequential CAR T cell and targeted alpha immunotherapy in disseminated multiple myeloma
Source: Cancer Immunol Immunother. 2023 May 20;72(8):2841–9. doi: 10.1007/s00262-023-03461-z (PMC10361855; doi:10.1007/s00262-023-03461-z)
Supplement: Supplementary file 1 — Supplementary file1 (DOCX 6470 kb) [file 262_2023_3461_MOESM1_ESM.docx]

**­­­Sequential CAR T cell and targeted alpha immunotherapy in disseminated multiple myeloma**

Dennis Awuah^1,^*, Megan Minnix ^2,^*, Enrico Caserta^3,^*, Theophilus Tandoh^3^, Vikram Adhikarla^4^, Erasmus Poku^5^, Russell Rockne^4^, Flavia Pichiorri^3^**, John E. Shively^2^** and Xiuli Wang^1,^**

**Table S1: Median Survival of therapy studies, days post MM1-S engraftment**.^1^

|  | **Control** | **Mock** | **CAR T** | **Mock + TAT** | **CAR T + TAT** | **CAR T + Tras** |
| --- | --- | --- | --- | --- | --- | --- |
| **Donor 1**  **3.7 kBq**  **14 days later**  **Fig. 1** | 40 | 49 | 49 | 71 | 71 | 49 |
| **Donor 2**  **3.7 kBq**  **14 days later**  **Fig. 2** | 48 | 44 | 96 | 61 | 89 | 75 |
| **Donor 2**  **7.4 kBq**  **29 days later**  **Fig. 3** | 47 | 59.5 | 68 | 71 | 106 | 94 |
|  |  |  |  |  |  |  |
|  | Control | TAT | TAT + CAR T 14 days later | TAT + CAR T  21 days later | TAT + CAR T  28 days later |  |
| **Donor 2**  **7.4 kBq**  **Fig. 4** | 42 | 68 | 91 | 91 | 77 |  |

^1^ Upper: For donor 1, Mock and CS1 CAR T gave equivalent survivals, therefore the experiment was repeated with a second donor where there was a difference in survival between Mock and CS1 CAR T. For donor 2, when 3.7 kBq TAT was delayed for 14 days, the survival was similar to CS1 CAR T monotherapy. Therefore, the study was repeated with a higher dose of TAT (7.4 kBq) and a delay of 29 days to minimize toxicity and to time TAT with tumor regrowth. Lower: The study was repeated with donor 2 with TAT (7.4 kBq) first followed by CS1 CAR T at 14, 21 or 28 days later. Controls were treated with injections of PBS. Mock= mock transduced T-cells. TAT= targeted α-therapy, UAT= untargeted α-therapy; Dara= daratumumab, Tras= trastuzumab.


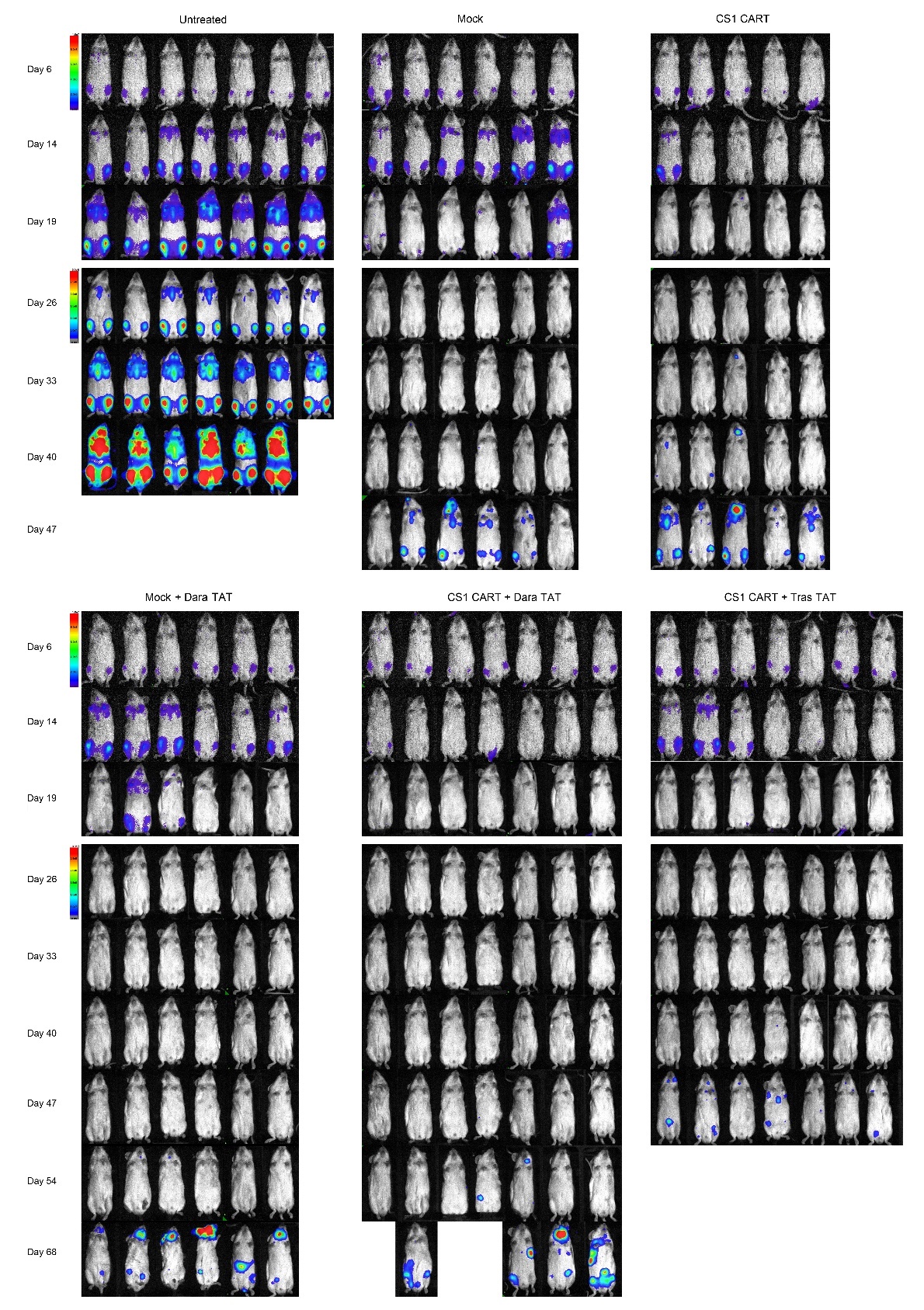


**Fig. S1. Images of all groups from Fig. 1.** Panels are of individual mice within each treatment group.


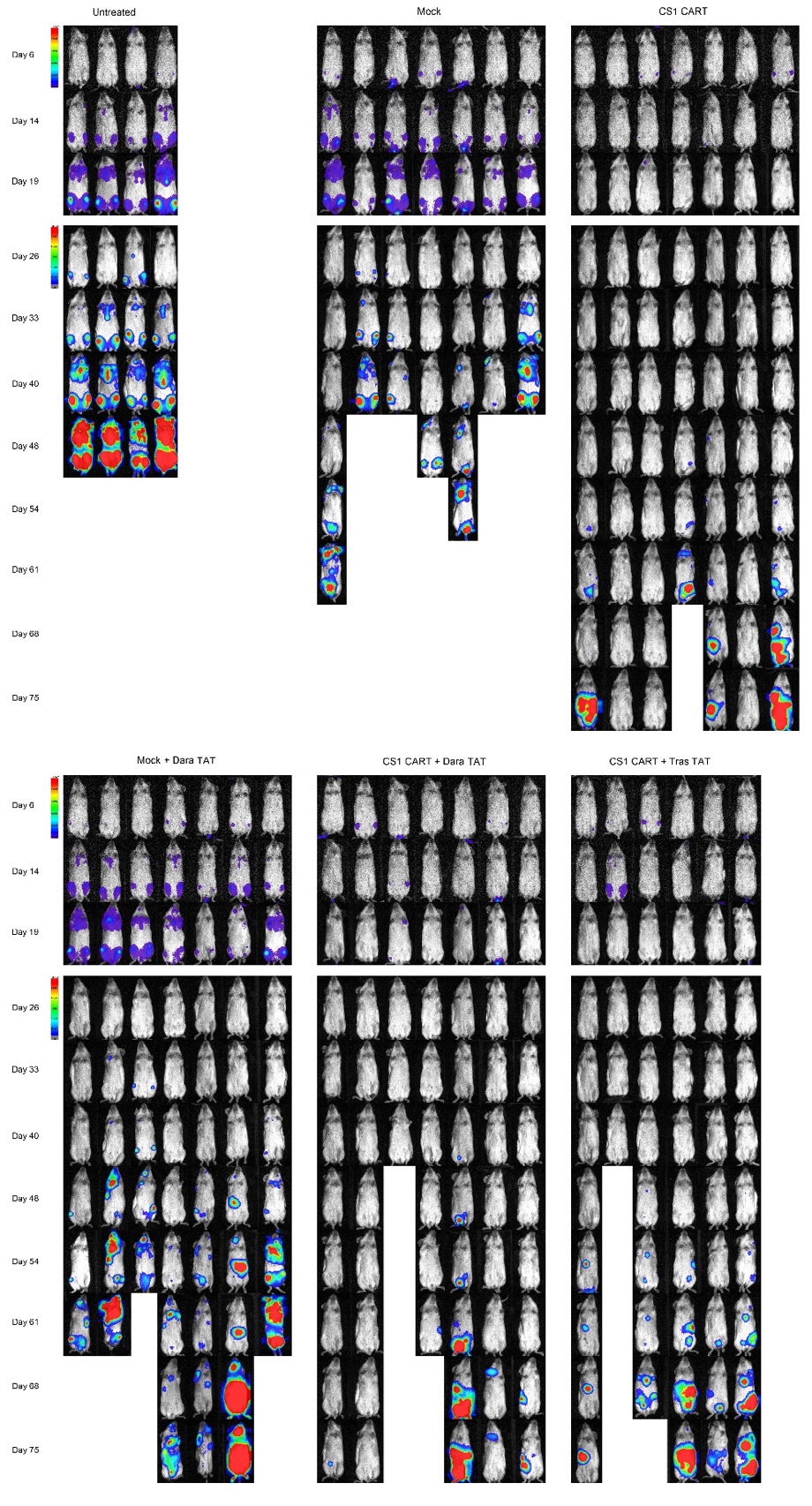


**Fig S2. Images of all groups from Fig. 2**. Sequential therapy with donor 2 CS1 CAR T and 3.7 kBq TAT 14 days later for treatment of disseminated MM. Panels are of individual mice within each treatment group.


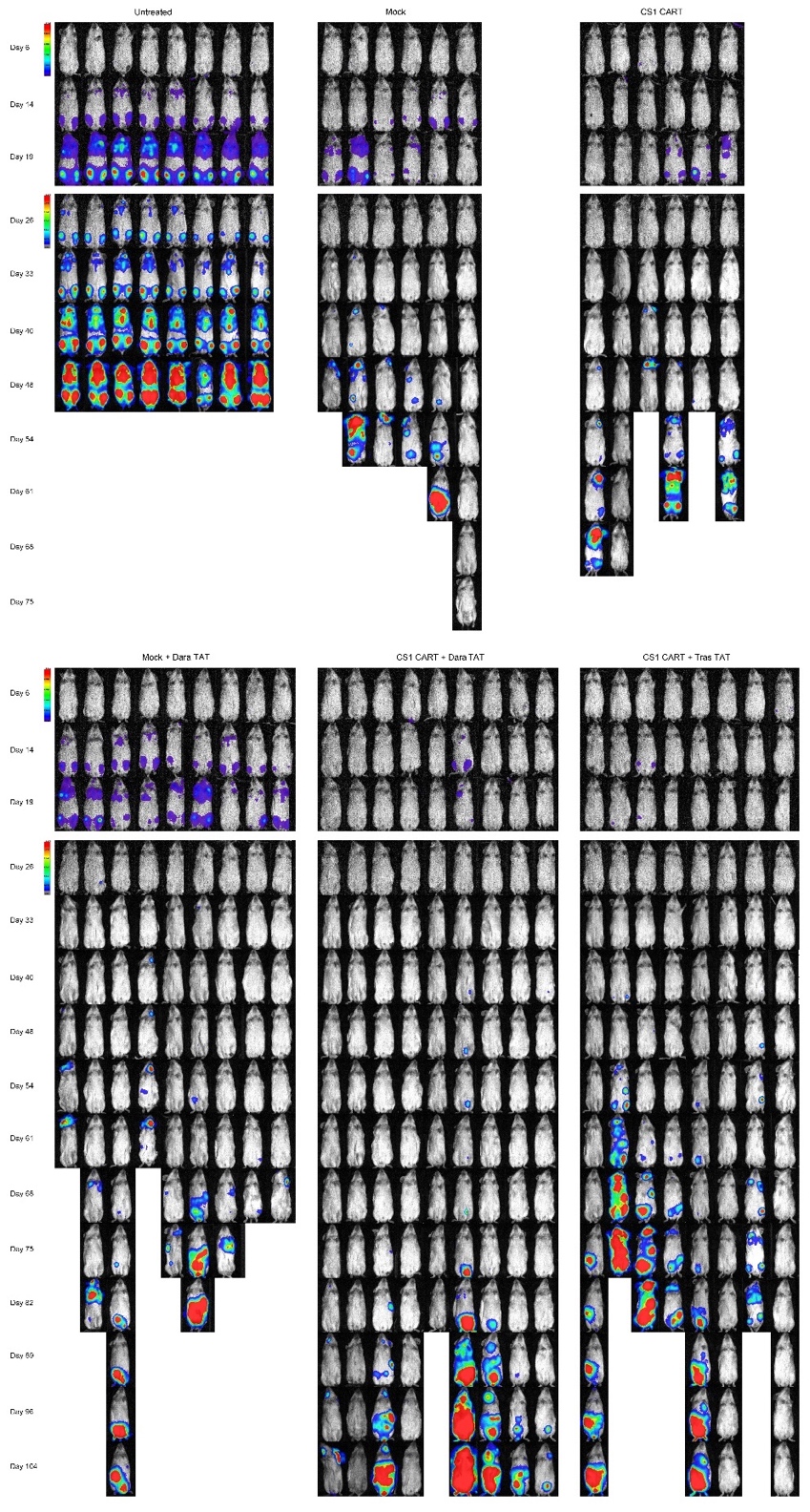


**Fig. S3. Images of all groups from Fig. 3**. Efficacy of sequential therapy with donor 2 CS1 CAR T and 7.4 kBq TAT 29 days later for treatment of disseminated MM. Panels are of individual mice within each treatment group.


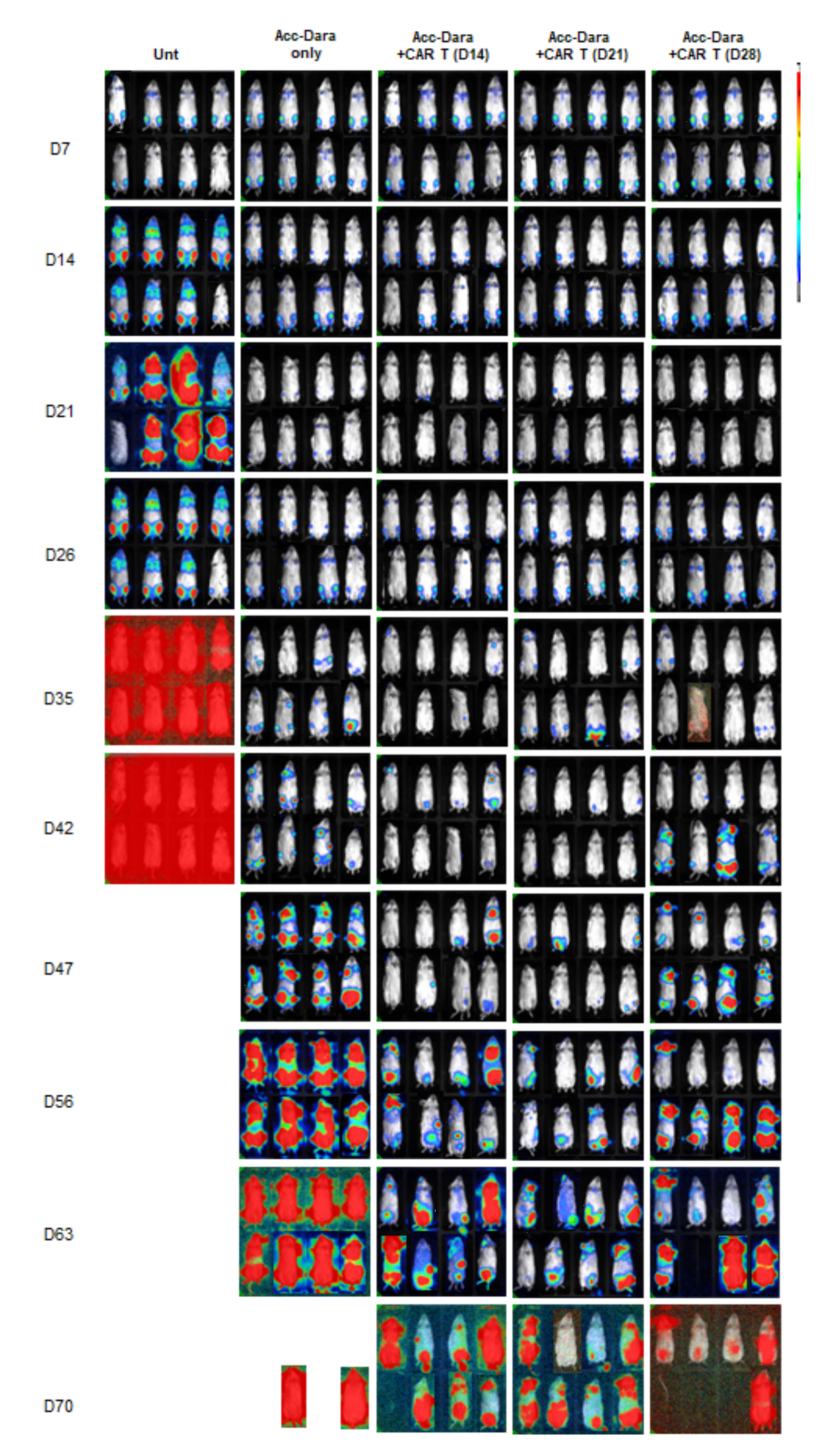


**Fig. S4. Images of all groups from Fig. 4**. Efficacy of sequential therapy with donor 2 with 7.4 kBq TAT followed by CS1 CAR T therapy at 14, 21 or 28 days later for treatment of disseminated MM. Panels are of individual mice within each treatment group.


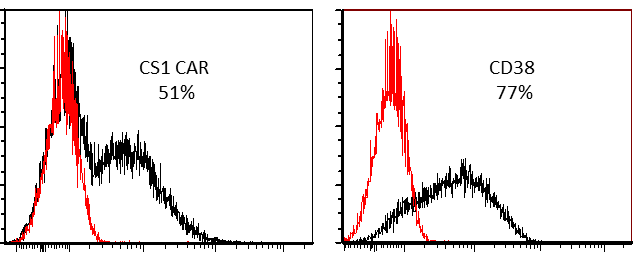


**Fig. S5. Expression levels of CS1 CAR and CD38 on CAR transduced T cells respectively**. Red histograms represent mock and black histograms represent markers of interest.
